# Natural Cellulosic Fiber Reinforced Concrete: Influence of Fiber Type and Loading Percentage on Mechanical and Water Absorption Performance

**DOI:** 10.3390/ma15030874

**Published:** 2022-01-24

**Authors:** Hafsa Jamshaid, Rajesh Kumar Mishra, Ali Raza, Uzair Hussain, Md. Lutfor Rahman, Shabnam Nazari, Vijay Chandan, Miroslav Muller, Rostislav Choteborsky

**Affiliations:** 1 Faculty of Textile Engineering, National Textile University, Faisalabad 37610, Pakistan; hafsa@ntu.edu.pk (H.J.); aliraza@ntu.edu.pk (A.R.); hussainuzirntu@gmail.com (U.H.); 2 Department of Material Science and Manufacturing Technology, Faculty of Engineering, Czech University of Life Sciences Prague, Kamycka 129, 165 00 Prague, Czech Republic; nazari@tf.czu.cz (S.N.); vchandan.2810@gmail.com (V.C.); muller@tf.czu.cz (M.M.); choteborsky@tf.czu.cz (R.C.); 3 Department of Textile, Primeasia University, Banani, Dhaka 1213, Bangladesh; Lutfor.shanzid@primeasia.edu.bd

**Keywords:** natural fiber, concrete, tensile, compression, bending, impact

## Abstract

The paper reports experimental research regarding the mechanical characteristics of concrete reinforced with natural cellulosic fibers like jute, sisal, sugarcane, and coconut. Each type of natural fiber, with an average of 30 mm length, was mixed with a concrete matrix in varying proportions of 0.5% to 3% mass. The tensile and compressive strength of the developed concrete samples with cellulosic fiber reinforcement gradually increased with the increasing proportion of natural cellulosic fibers up to 2%. A further increase in fiber loading fraction results in deterioration of the mechanical properties. By using jute and sisal fiber reinforcement, about 11.6% to 20.2% improvement in tensile and compressive strength, respectively, was observed compared to plain concrete, just by adding 2% of fibers in the concrete mix. Bending strength increased for the natural fiber-based concrete with up to 1.5% fiber loading. However, a decrease in bending strength was observed beyond 1.5% loading due to cracks at fiber−concrete interface. The impact performance showed gradual improvement with natural fiber loading of up to 2%. The water absorption capacity of natural cellulosic fiber reinforced concrete decreased substantially; however, it increased with the loading percent of fibers. The natural fiber reinforced concrete can be commercially used for interior or exterior pavements and flooring slabs as a sustainable construction material for the future.

## 1. Introduction

The construction industry around the world consumes huge volumes of non-renewable materials. This action not only causes wastage of minerals in millions of tons, but also the emission of carbon dioxide, which pollutes the environment [1,2]. In the early era, people used renewable resources to construct their homes and buildings. The use of renewable resources plays a vital role in eco-friendly infrastructure development in human society. Therefore, the construction industry is focusing on sustainable materials and techniques. The use of renewable resources in construction represents a major contribution in eco-friendly and sustainable development. The reinforcement of concrete with natural fibers is one of the most promising techniques to ensure strength improvement with non-hazardous effects on the environment. This also enables a sustainable and effective use of renewable resources [3,4,5]. The use of natural fiber reinforcement is gaining popularity in both industrial applications and for research purposes because natural fibers are renewable, inexpensive, and biodegradable. Their mechanical properties make them an attractive ecological alternative to synthetic fibers [6,7,8,9].

Portland cement concrete is brittle in nature and exhibits a relatively lower tensile strength, limited ductile properties, and limited resistance to cracking. Micro cracks exist inside the structure of concrete and result in a poor tensile strength because of crack propagation with time, and lead to brittle fractures. In plain Portland cement concrete and other similar brittle materials, the shrinkage during drying also causes micro cracks. When load is applied on these materials, the micro cracks propagate, and the size of cracks gradually increases causing an inelastic deformation in the concrete material. Therefore, the addition of small size natural fibers in concrete, which are uniformly distributed and randomly oriented, can reduce the drying shrinkage by arresting the propagation of micro cracks and at the same time increasing the strength. This addition of fibers in concrete is known as fiber reinforcement of concrete. The addition of steel as a reinforcement material in concrete reduces the formation of micro cracks while improving the mechanical performance. However, with the passage of time, steel gets rusted due to various oxidative reactions. On the other hand, the reinforcement of renewable natural fibers in concrete is an environment friendly approach and incurs lower production costs [10,11,12]. Several researchers have investigated about the addition of natural sustainable fibers, e.g., jute, hemp, pineapple, basalt, sisal, and banana, in concrete and observed an improvement in mechanical properties and also a reduction in crack propagation inside the concrete [12,13,14,15]. The reported results reveal that through the addition of natural fibers, some properties may be enhanced and some may be deteriorated [13,14,15,16]. The addition of natural fibers of up to 1% of the cement mass improved the compressional properties in concrete, and the sudden brittle failures are restricted with the increasing percentage of fibers [17,18,19].

Several researchers reported findings on the reinforcement of jute fibers in concrete in order to increase the compressive, bending, and impact strengths [20,21,22]. The results reveal that the properties improve through the reinforcement of the jute fibers up to certain limit. The technique for the extraction of inert carbonized particles from bamboo and cement composites and the effects of their addition have been reported [23]. The results revealed that the flexural strength and the toughness of cement composites increase through the addition of bamboo particles. Researchers have worked on the reinforcement of basalt fibers in high performance concrete and have done experimentation to check their tensile strength, modulus, and compressive strength, etc. [24]. It was observed that the compressive strength increases by increasing fiber volume percentage up to 2%. Above 2% fiber loading, the strength started to decrease. Basalt fiber volume percentages had no significant effect on the elastic modulus values [24]. Several researchers investigated different reinforcement possibilities of coconut fibers and ropes in concrete to cast columns [25,26]. The aim was to produce safe housing in earthquake prone areas. Owing to having the highest toughness among all natural fibers, coconut fibers were extensively used in the concrete. The impact tests of fiber and rope reinforced concrete columns were carried out and it was found that the column cast with ropes was better compared to the others [25,26]. A superior mechanical performance was observed in the case of concrete reinforced with kenaf fibers compared to conventional plain concrete samples [27]. Additionally, the results revealed that fiber-reinforced concrete generally disperse cracking and exhibit more toughness than the plain concrete samples.

The fracture energy of natural fiber reinforced concrete using elephant grass, hemp, and wheat straw has been reported [28]. Concrete samples containing 0.19% weight of 40 mm long fibers were tested uniaxially using the wedge splitting test (WST) method. The fracture toughness improved with the addition of fibers to plain concrete. An up to 70% increase in the fracture energy was observed in hemp fiber reinforced concrete compared to the plain concrete samples. A moderate increase of about 2% and 5% was observed in straw and elephant grass reinforced concrete, respectively. Several locally available materials were used to produced high strength concrete (HSC) [28,29,30]. Researchers analyzed the effect of 10–30% rice husk ash (RHA) reinforcement in cement on the tensile strength. Rice husk, an agricultural waste material, is abundantly available in developing countries. The compressive and split tensile strengths of the samples were analyzed. When cement was partially replaced by RHA for maintaining the same level of workability, the strength of the HSC decreased. Many researchers have recently studied various types of textile-based reinforcement in concrete and have investigated the interfacial bond between fibrous reinforcement and concrete using the fiber pull-out test [31,32,33]. The accelerated ageing test caused the degradation in textiles composed of jute and polyester fibers, while basalt and polypropylene (PP) fiber-based textiles resisted their degradation in an alkaline environment. The results showed that basalt fiber reinforced concrete shows superior mechanical properties than the other natural origin fibers investigated [34,35,36,37]. There have been numerous attempts to use natural fibers in pure and hybrid form as a reinforcement in composites and concrete [38,39,40]. Several experimental and theoretical research works have been reported that demonstrate the advantage of using fiber-based reinforcement in concrete [41,42].

The current research is focused on an experimental investigation of the mechanical characteristics in natural origin sustainable cellulosic fiber-reinforced concrete. Jute, sugarcane, sisal, and coconut fibers with average length of 30mm were mixed with concrete matrix in varying percentages (0.5–3%) of cement mass. The results were also compared with the control samples (without fiber reinforcement) and the improvement in mechanical properties by adding natural fibers was quantified. Statistical analysis was carried out using design of experiment (DoE) and analysis of variance (ANOVA) methods in order to quantify the significance and the contribution of experimental parameters on the mechanical and water absorption properties in concrete samples reinforced with natural cellulosic fibers.

## 2. Materials and Methods

### 2.1. Materials

The constituents of natural cellulosic fiber-reinforced concrete investigated in this work are ordinary Portland cement (OPC), gravels/aggregates, fine sand, and selected natural fibers. Different varieties of natural cellulosic fibers were used for the preparation of the concrete samples. Jute fiber was selected from the category of bast fibers. Among the leaf fibers, sisal was selected. From the category of fruit fiber, coconut/coir fibers, and from cane fiber category, sugarcane bagasse, were chosen. Bagasse is a waste product of the sugar cane industry. All these fibers show a high strength/weight ratio as well as a high aspect ratio. Coconut/coir and jute fibers were obtained from Pakistan, sisal fiber was purchased from China, and sugarcane bagasse fibers were manually extracted from sugarcane as reported in the literature [43,44,45,46,47].

Apart from sugarcane, all other fibers were procured in finished form. Sugarcane bagasse was obtained from the agricultural waste by a manual scutching process. After the juice was squeezed/extracted from the plant, the fibrous stem was disposed as waste. These stems were collected, thoroughly washed, and then dried. The fibers were individualized by a scutching/beating operation. All the fibers were cut into 30 ± 2 mm length. The jute, sisal, and coconut/coir fibers were procured from the market. However, the properties were evaluated in the laboratory. Some basic properties were taken from the literature [44]. All of the fibers were conditioned at 25 °C and 65% RH for 48 h before testing and characterization. The fiber fineness was measured using ASTM D2252-18 standard [48]. The fiber linear density was measured using ASTM D7025-09 [49], fiber length was measured using ASTM D5103-07 [50], fiber aspect ratio was evaluated by ASTM A820M [51], density was measured using ASTM D8171-18 [52], porosity was estimated using ASTM D3171-15 [53], and fiber mechanical properties were evaluated using ASTM D3822M-14 [54].

The chemical ingredients of ordinary Portland cement (OPC), as obtained from the supplier, are given in Table 1.

The detailed characteristics of the natural fibers utilized are presented in Table 2. The physical/mechanical properties were measured using well known ASTM standards and other parameters, e.g., cellulose content, lignin content, crystallinity, and orientation angle, were taken from the literature [44].

### 2.2. Methods

**Figure 1:**
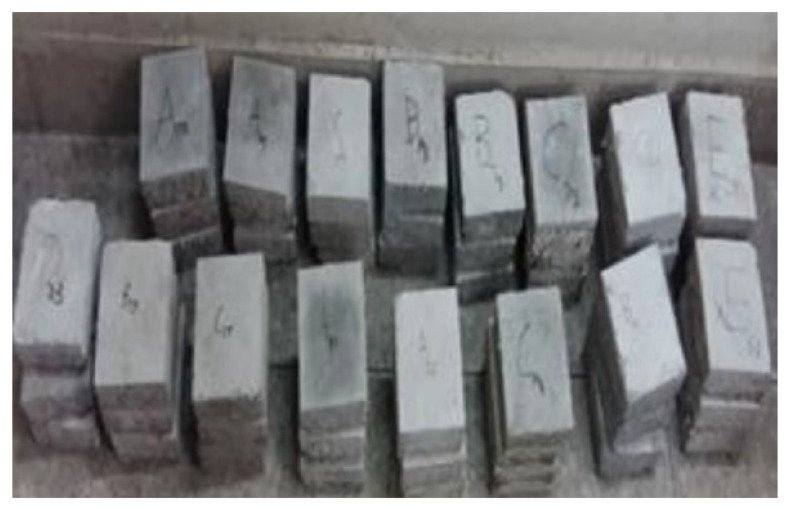
Cured concrete samples with natural fiber reinforcement.

In order to prepare the concrete samples with cellulosic fiber reinforcement, a 1:2:4 volume ratio of cement, sand, and gravel was maintained. The water/cement proportion of 0.55 by mass was kept for all samples [20]. The natural cellulosic fibers in various ratios were thoroughly mixed with cement to make a homogenous blend of cement, gravel, sand, and fibers. The mixture was casted into mold of cubic sizes (5.2 cm × 5.2 cm × 5.2 cm) for compression and absorption testing, cylindrical sizes (D = 7.4 cm and L = 13 cm) for tensile testing, rectangular (13 cm × 2.56 cm × 2.56 cm) for bending testing, and rectangular (10.2 cm × 15.4 cm × 2.56 cm) for impact testing. Samples of pure concrete (control sample) were made for each test with the same dimensions for a comparison of their mechanical properties. All of the samples were prepared as per the ASTM standard. Before testing of the prepared samples, almost one month was given to the samples for curing. The cured samples are shown in Figure 1.

Microscopic analysis was done for the fibers using scanning electron microscopy (SEM, Tescan Orsay Holding, a.s., Brno, Czech Republic) and for the fracture surface of concrete samples after mechanical testing using light microscopy. The samples were prepared with a sputter named Quorum Q150R ES (Quorum, Laughton, East Sussex, UK), using gold-plating in an argon gas atmosphere. The thickness of the gold plating was 2 nm, and a current of 20 mA was used. A magnification of 10,000× was used for SEM and 100× was used for analyzing the fracture surface of the samples [42].

**Figure 2:**
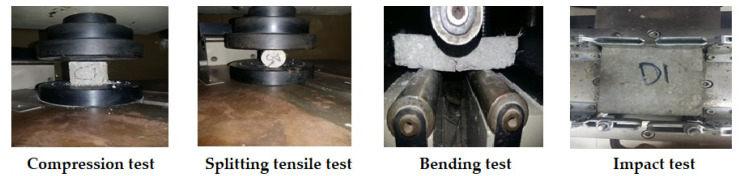
Principles of mechanical testing in concrete.

In order to check the mechanical performance and durability of the natural cellulosic fiber reinforced concrete samples as compared to plain concrete, the splitting tensile strength, bending strength, compressive strength, impact test, and water absorption tests were conducted. The mechanical test principles are shown in Figure 2.

The splitting tensile testing of concrete samples with a cylindrical shape (diameter = 7.4 cm and length = 13 cm) was determined by Digital-Display Hydraulic Universal Testing Machine (Beijing Sinofound Co. Ltd., Beijing, China) with testing standard ASTM C496-96 [55]. The loading was carried out at a speed of 1 mm/min. Splitting tensile strength was calculated as follows:

(1) $$S_{t}=\left[\frac{2P}{\pi DL}\right]$$

where *S_t_* = splitting tensile strength, *P* = pressure for cracking, *D* = diameter of specimen, and *L* = length of specimen.

To measure the bending strength of the samples, rectangular concrete blocks (13 cm × 2.56 cm × 2.56 cm) were made as per the ASTM standard. The Hydraulic Universal Testing Machine (Model: Beijing Sinofound WES-100) was used to measure the value of the bending strength. The test standard ASTM C78/C78M-21 was used [56]. A loading speed of 1 mm/min was used. The bending strength was calculated as below.

(2) $$\sigma =3PL/2bh^{2}$$

where *P* represents load, *L* represents gauge length, *b* represents width, and *h* represents thickness. Ten measurements were carried out and the average was reported.

The compressive testing of natural fiber reinforced samples and plain concrete samples of cubic size (5.2 cm × 5.2 cm × 5.2 cm) were determined by a Digital-Display Hydraulic Universal Testing Machine (Model: Beijing Sinofound WES-100) with testing standard ASTM C109/C109M-07 [57]. The loading speed was 1 mm/min. Compression strength was obtained from the device directly. An average of 10 measurements was reported.

The impact strength was measured using rectangular blocks (size 10.2 cm × 13.4 cm × 2.56 cm). The drop weight impact tester (Model: HIT230F, Zwick Roell group, Ulm, Germany) was used to measure the energy absorbed by the samples according to the ASTM C1747_C1747M-13 standard [58]. A maximum energy of 10J was applied on the samples through the drop weight impact tester and the absorbed energy was calculated.

Impact energy absorbed (*E*) = *M* × *g* × *h* (3)

where *M* = mass of impactor, *g* = acceleration due to gravity, and *h* = drop height of the impactor.

Ten measurements were conducted for each type of mechanical property and the mean was reported with coefficient of variation (CV% < 5%). All results were considered for calculation of the mean. In case some results were out of scale (CV > 5%), the measurements were repeated.

For theoretical estimation of mechanical properties of the fiber-reinforced concrete, the corresponding mechanical properties of the fibers and pure concrete were evaluated experimentally. The mechanical properties of the cellulosic fiber reinforced concrete were estimated using Halpin–Tsai model. This model is commonly used for prediction of the effective mechanical properties of fiber-reinforced concrete (composite) with well-defined fiber alignment [33,34,35]. The Halpin–Tsai equation is expressed as:

(4) $$K_{c}=K_{m}\left[\frac{1+\xi \zeta V_{f}}{1-\eta V_{f}}\right]$$

(5) $$\mathrm{With}\;\eta =\left[\frac{\left(K_{f}/K_{m}\right)-1}{\left(K_{f}/K_{m}\right)+\zeta }\right]$$

where *K_c_* = effective mechanical property of the fiber reinforced concrete; *K_f_* and *K_m_* are the corresponding fiber and concrete mechanical properties, respectively; *V_f_* = the fiber volume fraction; and *ζ* = geometrical parameter, which represents the loading conditions (e.g., uniaxial, biaxial, and multiaxial).

In order to measure the water absorption capacity (%) of fiber reinforced concrete samples comparative to plain concrete samples, blocks of cubic size 5.2 cm × 5.2 cm × 5.2 cm were prepared. The water absorption (%) was checked on Specific Gravity apparatus (Humboldt Mfg. Co., Elgin, Scotland) with electronic balance using the ASTM C127-04 testing standard [59]. A mean value of 10 measurements was reported.

For statistical analysis, full factorial design of experiment (DoE) was used [27]. Table 3 shows the experimental design (DOE) for the samples developed.

Analysis of variance was used to check the significance of the experimental parameters affecting the mechanical and durability functions of the concrete samples at the 95% confidence interval. Minitab 17 software was used for this purpose.

## 3. Results and Discussion

### 3.1. Microscopic Analysis of Fiber Crosssection

**Figure 3:**
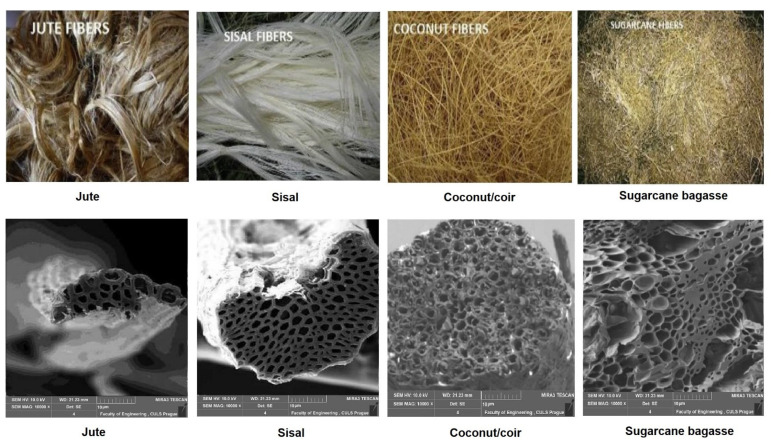
Microscopic images of the cellulosic fibers used.

Cross-sectional images of the fiber samples were taken using scanning electron microscopy (SEM), as shown in Figure 3.

It can be observed that the four types of fibers used in this study contained a large amount of air due to the pores in their microstructures. As compared to jute and sisal, the coir and bagasse fibers showed a higher porosity. They had a higher diameter and thus a more open structure.

### 3.2. Compressive Strength

**Figure 4:**
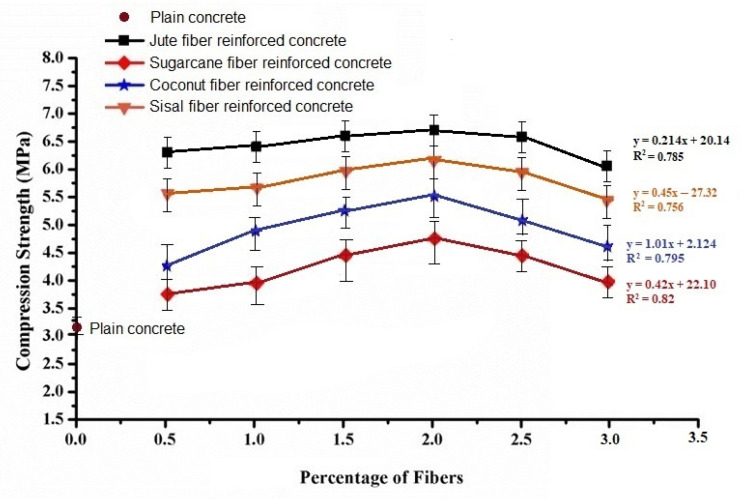
Variations of compressive strength with different fiber loading percent.

Figure 4 presents changes in the compressive strength of natural cellulosic fiber-reinforced concrete samples with different fiber loading percent.

**Figure 5:**
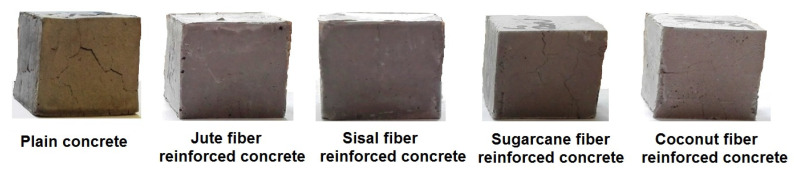
Sample concrete blocks after compression.

For all the fiber types (coconut, sugarcane bagasse, jute, and sisal) of reinforced concrete samples, the compressive strength gradually increased with the increase in percentage of natural fibers in concrete up to 2% fiber loading. Beyond this limit, the compressive strength further decreased. In the case of 0.5% jute and sisal fiber reinforcement, an increase of 11.6% to 20.2% in compressive strength was observed compared to plain concrete. Furthermore, 0.5% coconut fiber reinforcement in concrete increased the compressive strength by about 9%. Increasing the fiber loading up to a maximum of 2% resulted in an increase of the compressive strength in natural fiber reinforced concrete samples. Such observations are based on the rule of mixture and have been reported by several other researchers [6,7,8,9]. A further increase in fiber loading seemed to generate voids and thus may be a reason for fracture during compression loading. The sample concrete blocks with 2% fiber loading, which resulted in a maximum compressive strength, are shown in Figure 5.

The differences in behavior of concretes with different types of cellulosic fibers follow similar trend as the respective fiber mechanical properties. As the jute and sisal fibers have inherently superior mechanical properties compared to coconut and sugarcane, the same effect is reflected in the concrete samples reinforced by the respective fibers. This trend is consistent for all the different fiber loadings up to 2%. As per Halpin−Tsai model the mechanical performance depends on the fiber and matrix (concrete) mechanical properties and fiber volume fraction. In case of higher fiber loadings, the decreasing trend may be attributed to irregularities in fiber orientation and the presence of voids due to a higher fiber percentage.

### 3.3. Splitting Tensile Strength

**Figure 6:**
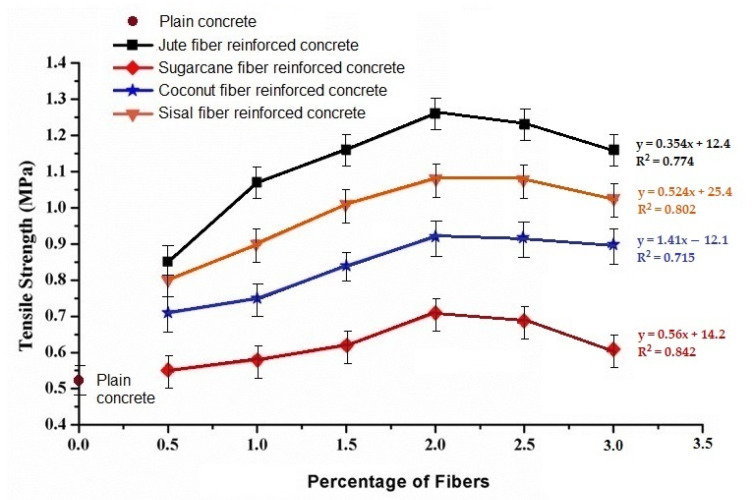
Variation of tensile strength with different fiber loading%.

Figure 6 presents a summary of tensile strength of the natural cellulosic fiber-reinforced concrete with different fibers and corresponding loading percentages.

For all the natural fiber-reinforced concrete samples, the tensile strength gradually increased compared to the control sample, with an increasing fiber loading percent up to 2%. The increase in tensile strength by varying quantity of natural fibers for jute, sisal, sugarcane, and coconut fiber reinforced concrete samples is 60.4% to 137.7%, 51% to 103.8%, 3.8% to 34%, and 34% to 73.6%, respectively. The tensile strength of fiber-reinforced concrete depends on the tensile strength of the reinforcing fibers. The tensile strength and tenacity of the fibers is in the order jute > sisal > coconut > sugarcane. A similar trend in the concrete samples reinforced by such fibers is observed. The observations are confirmed by theoretical calculation of the tensile properties based on the Halpin−Tsai model, as given in Table 4.

**Figure 7:**
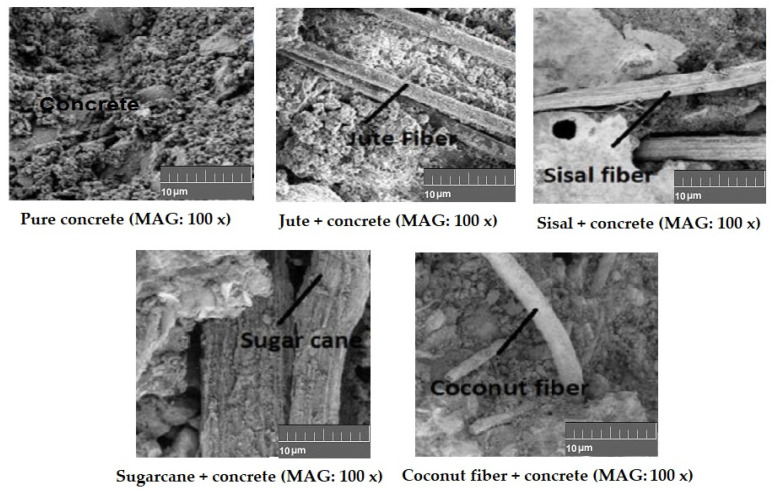
Interfacial bonding between different natural fibers and concrete.

The microscopic images shown in Figure 7 indicate the interfaces between natural fibers and concrete matrix.

However, it was observed that the tensile strength further decreased as the fiber loading increased to 2.5% and 3%. These findings were along the same line as the compression properties. It might therefore be concluded that a fiber loading higher than 2% results in a decrease in strength of natural fiber-reinforced concrete. This is again attributed to irregularities in fiber distribution and resulted in voids in the concrete.

### 3.4. Bending Strength

**Figure 8:**
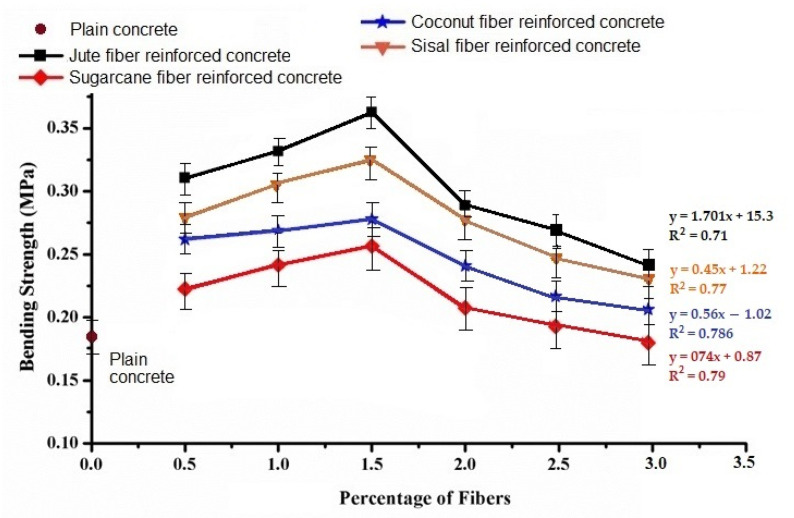
Variation of bending strength with different fiber loading percent.

The results in Figure 8 show the bending behavior of natural fiber-reinforced concrete samples with varying fiber types and loading percent.

**Figure 9:**
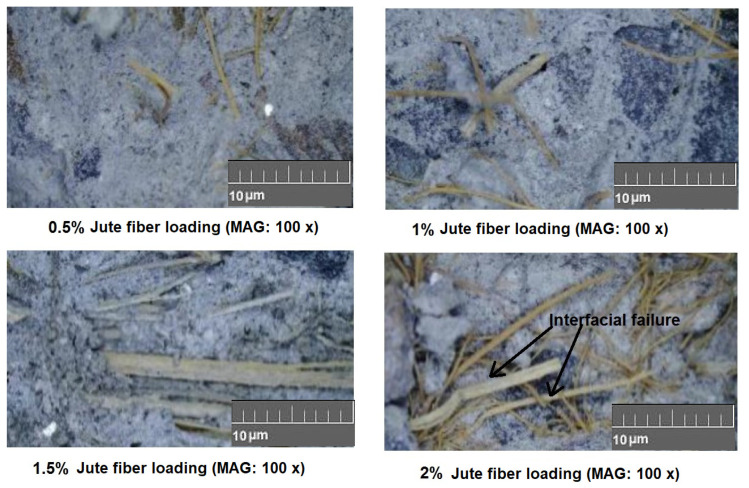
Interfacial failure after the bending test for different fiber loading percent.

Bending strength increased with increasing the loading percent of fibers up to 1.5%. Increasing the fiber loading beyond 1.5% in concrete caused a visible decrease in the bending strength. Concrete samples reinforced with jute and sisal fibers exhibited a superior bending strength as compared to the coconut and sugarcane fiber reinforced samples. The bending properties of fiber-reinforced concrete also depended on the tensile properties of the fibers. Thus, an increase in bending strength was clearly based on the fiber tensile properties. Up to 1.5% fiber loading, the Halpin−Tsai equations predicted the bending performance. The results are given in Table 4. The subsequent decrease in bending property upon the increase of fiber loading beyond 1.5% could be due to the increase in porosity and weak spots at the fiber−concrete interface. However, the overall bending strength for reinforced concrete at all fiber loading percent was always higher than for the plain concrete sample. The obtained results are similar to results reported by other researchers in literature [6,7,8,9]. The damaged samples after the bending test were analyzed by optical images of cracks. The nature of the crack showed that fiber loading higher than 1.5% was prone to crack initiation at the interfacial regions. The images of the jute fiber-reinforced concrete samples (which show the highest bending strength among all samples) after cracking are shown in Figure 9.

### 3.5. Impact Energy Absorbed

**Figure 10:**
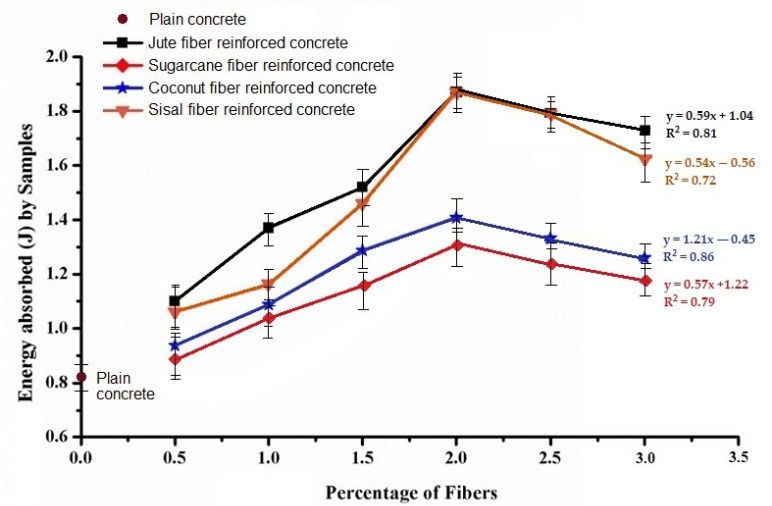
Energy absorbed (J) with different percentages of fiber.

Figure 10 shows the impact energy absorbed by the natural fiber-reinforced concrete samples comparative to the plain concrete. The samples that absorbed more energy comparative to others had a higher impact strength. The results reveal that the energy absorbed by natural fiber-reinforced concrete samples gradually increased with the increase in percentage of fiber loading up to 2%. The fiber reinforced samples showed a significantly higher impact strength compared to the plain concrete.

These observations support several previous researchers [6,7,8,9]. The maximum energy (J) absorbed was for the jute fiber-based samples, which varied between 1.1 J to 1.87 J, while sisal-, sugarcane-, and coconut-based concrete samples absorbed energy amounting 1.06 J to 1.84 J, 0.84 J to 1.31 J, and 1.02 J to 1.44 J, respectively with corresponding percentage of fibers from 0.5% to 2%. The increase in impact strength by varying the quantity of natural fibers for jute, sisal, coconut, and sugarcane fiber reinforced concrete samples was 60.4% to 137.7%, 51% to 103.8%, 34% to 73.6%, and 3.8% to 34%, respectively. The fiber reinforcement enhanced the impact performance of the concrete, as fibers can absorb a higher impact energy than pure concrete. The internal geometry of fibers that are composed of amorphous and crystalline polymer segments facilitates the absorption of the impact energy. The Halpin−Tsai models support such observations [31,32,33,34,35,36,37]. This increasing trend is observed up to 2% fiber loading only, and with the subsequent increase of fiber percent, the impact energy goes down. These observations are supported by previous results of compression and tensile strength. An increase of fiber loading beyond 2% resulted in irregularities and imperfections in the concrete, which could be sites of crack initiation and thus result in a lower impact energy.

### 3.6. Water Absorption Test

**Figure 11:**
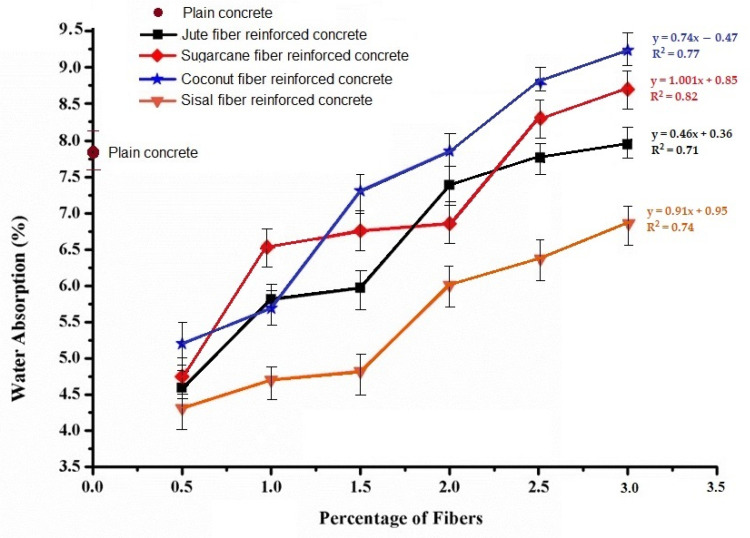
Water absorption capacity (%) with different percentages of fiber loading.

Figure 11 shows a summary of the water absorption capacity of the natural cellulosic fiber-reinforced concrete samples with varying fiber types and different percentage loading of fibers. The results are presented with reference to the control sample (pure concrete). Generally, the fiber-reinforced concretes exhibit a more reduced absorption capacity than plain concrete, especially at a lower fiber loading percent. Lower water absorption increased the durability and stability of concrete.

For all the natural fiber (jute, sisal, sugarcane, and coconut) reinforced concrete samples, the capacity of water absorption increased with a higher fiber loading percentage. The overall water absorption capacity of the natural cellulosic fiber-reinforced concrete varied in the range of 4.25% to 9.25% by changing the fiber loading percentage from 0.5% to 3%. As observed from the fiber−concrete interface images in Figure 7 and Figure 9, the porosity at the interface increased as the fiber loading increased. Thus, the water absorption capacity increased as the fiber loading increased from 0.5% to 3%. Among the different types of natural fibers used in this investigation, coconut and sugarcane fibers had a higher porosity and lower crystallinity compared to the jute and sisal fibers. Therefore, the concrete samples reinforced with these types of fibers showed a relatively higher water absorption capacity.

### 3.7. Statistical Analysis Using ANOVA

The analysis of variance was used to find the significance of the natural fiber type and loading percent on the mechanical and durability/water absorption performance of concrete. Table 5 shows the contribution (%) of the experimental factors on the mechanical and durability performance of natural cellulosic fiber-reinforced concrete.

The ANOVA results show that experimental factors like type of natural fiber reinforcement and the loading of fiber have a significant influence on several aspects of the mechanical performance of concrete, e.g., compression, tensile, bending, and impact strength. Furthermore, the type and amount of fiber loading has a significant influence on the water absorption capacity.

## 4. Conclusions

The mechanical performance of natural cellulosic fiber-reinforced concrete was observed to improve up to a certain limit for fiber loading. With a further increase in the loading percent of natural fibers, there is decrease in tensile, bending, compressive, and impact performance. Such behavior at a fiber loading higher than 2% is attributed to cracks and voids due to irregular fiber orientation, and thus the initiation of the mechanical failure in concrete.

The results reveal that the compressive strength of natural fiber-reinforced concrete increases with fiber loading up to 2%. For jute and sisal fiber reinforcement, about a20.2% and 11.9% improvement in compressive strength, respectively, was observed compared to the plain concrete.

The tensile strength of natural fiber-based concrete gradually increases with the increase in percentage of natural fibers in concrete. The increase in tensile strength for jute-, sisal-, coconut-, and sugarcane fiber-based samples is up to 137.7%, 103.8%, 73.6%, and 34%, respectively by varying percentages of fibers up to 2%.

While the bending strength of the natural fiber reinforced concrete samples increased by increasing the fiber loading until 1.5%, there was a decrease beyond 1.5%. At 1.5%, the bending strength was observed to be at a maximum for the jute fiber reinforced concrete sample (0.369 MPa) followed by sisal-based concrete (0.291 MPa), coconut (0.254 MPa), and sugarcane fiber reinforced concrete (0.246 MPa).

The energy absorbed by the fiber reinforced concrete increased with the increase in percentage of natural fibers up to 2%, and hence the impact strength increased. The maximum energy (J) absorbed was observed for jute fiber-based concrete, which was 1.87 J followed by sisal, coconut, and sugarcane-based samples, which absorbed energy of 1.84 J, 1.44 J, and 1.31 J respectively.

In the natural fiber reinforced concrete, the water absorption capacity gradually increases with the increase in percentage of fibers. The inter fiber and interfacial porosity of coconut- and sugarcane fiber-based concrete enables a higher water absorption compared to jute and sisal fiber-based concrete.

The natural cellulosic fiber-reinforced concrete offers numerous scopes for the replacement of pure concrete in slabs and pillars. Thus, a sustainable building material with an enhanced mechanical and durability performance can be commercially produced. Furthermore, the material can be made more economical by using agricultural and industrial waste fibers.

## Figures and Tables

**Table 1:** Chemical ingredients of ordinary Portland cement (OPC).

Ingredient	Minimum%	Average%	Maximum%
SiO_2_	18.4	21.02	24.5
SO_3_	0	2.58	5.35
Fe_2_O_3_	0.16	2.85	5.78
CaO	58.1	64.18	68
MgO	0.02	1.67	7.1
Al_2_O_3_	3.1	5.04	7.56
K_2_O	0.04	0.7	1.66
Na_2_O	0	0.24	0.78
Free lime	0.03	1.24	3.68

**Table 2:** Detailed characteristics of the natural cellulosic fibers used.

Fiber Characteristics	Jute Fiber	Sisal Fiber	Sugarcane Bagasse Fiber	Coconut/Coir Fiber
Fiber diameter (µ)	18 ± 1.1	20 ± 1.2	22 ± 1.2	21 ± 1.1
Fiber fineness (Tex, g/km)	17 ± 1.1	21 ± 1.2	32 ± 1.2	30 ± 1.3
Fiber length (mm)	30 ± 2	30 ± 2	30 ± 2	30 ± 2
Fiber aspect ratio (-)	167–344	200–400	136–318	143–429
Density (g/cm^3^)	1.3	1.5	0.82	1.2
Porosity (%)	15–17	12–14	39–42	32–35
Cellulose content (%)	61–72	66-78	45–55	32–43
Lignin content (%)	12–13	8–11	19–24	41–45
Crystallinity (%)	60–65	68–70	51–53	27–33
Angle of orientation (°)	8–10	10–25	14–15	30–49
Tensile strength (MPa)	480 ± 16.2	381 ± 23.6	68 ± 9.1	175 ± 8.2
Elongation at break (%)	2.3 ± 0.1	2.45 ± 0.1	1.5 ± 0.1	3.6 ± 0.2
Modulus (GPa)	37.5 ± 1.4	28.5 ± 0.8	18.7 ± 0.8	22.0 ± 0.2
Tenacity (cN/Tex)	20.02 ± 1.5	17.7 ± 0.9	14.2 ± 0.4	15.3 ± 0.6

**Table 3:** Experimental design (DoE).

Sample No.	Reinforcement Type	% Fiber Loading
1	None	0%
2	Jute	0.5%
3	Sisal	0.5%
4	Sugarcane	0.5%
5	Coconut	0.5%
6	Jute	1%
7	Sisal	1%
8	Sugarcane	1%
9	Coconut	1%
10	Jute	1.5%
11	Sisal	1.5%
12	Sugarcane	1.5%
13	Coconut	1.5%
14	Jute	2%
15	Sisal	2%
16	Sugarcane	2%
17	Coconut	2%
18	Jute	2.5%
19	Sisal	2.5%
20	Sugarcane	2.5%
21	Coconut	2.5%
22	Jute	3%
23	Sisal	3%
24	Sugarcane	3%
25	Coconut	3%

**Table 4:** Predictions of the Halpin−Tsai model and the experimental results.

Sample No.	Tensile Strength (MPa) Predicted by Halpin-Tsai Model	Experimental Tensile Strength (MPa)	Bending Strength (MPa) Predicted by Halpin-Tsai Model	Experimental Bending Strength (MPa)
1	0.52	0.52	0.18	0.18
2	0.85	0.84	0.32	0.31
3	0.81	0.80	0.30	0.27
4	0.56	0.55	0.24	0.22
5	0.71	0.69	0.29	0.26
6	1.08	1.05	0.35	0.33
7	0.88	0.87	0.33	0.29
8	0.57	0.56	0.27	0.23
9	0.74	0.72	0.31	0.26
10	1.16	1.14	0.39	0.36
11	0.97	0.95	0.36	0.31
12	0.61	0.59	0.30	0.24
13	0.84	0.80	0.36	0.27
14	1.28	1.25	0.42	0.29
15	1.01	0.99	0.40	0.27
16	0.65	0.62	0.34	0.21
17	0.88	0.85	0.41	0.24
18	1.33	1.21	0.48	0.27
19	1.08	0.96	0.43	0.25
20	0.68	0.61	0.38	0.20
21	0.95	0.84	0.46	0.23
22	1.38	1.14	0.54	0.26
23	1.10	0.93	0.48	0.24
24	0.71	0.58	0.41	0.19
25	1.01	0.83	0.51	0.21

**Table 5:** Analysis of variance for performance of concrete.

ANOVA Terms	Compression Strength	Tensile Strength	Bending Strength	Impact Energy	Water Absorption Capacity %
R-sq (predicted) %	92.42	93.23	85.35	91.78	85.14
Sum of squares (SS)	5.54	5.86	5.07	5.82	4.23
Mean sum of squares (MS)	2.25	2.01	2.78	2.16	2.12
F-statistic	1.01	1.11	1.03	1.1	1.2
Error%	4.41	3.20	5.44	4.71	4.88
% contribution of reinforcement fiber type	54.23	60.74	42.86	46.28	69.14
% contribution of fiber loading	45.77	39.26	57.14	53.72	30.86
*p* value					
Type of fiber reinforcement	*p* ≤ 0.001	*p* ≤ 0.012	*p* ≤ 0.000	*p* ≤ 0.001	*p* ≤ 0.002
Fiber loading%	-------	-------	*p* ≤ 0.002	*p* ≤ 0.535	*p* ≤ 0.005

## Data Availability

Data sharing not available.
